# Overexpression of Replication-Dependent Histone Signifies a Subset of Dedifferentiated Liposarcoma with Increased Aggressiveness

**DOI:** 10.3390/cancers13133122

**Published:** 2021-06-22

**Authors:** Yongjin Yoo, Sang-Yoon Park, Eun Byeol Jo, Minji Choi, Kyo Won Lee, Doopyo Hong, Sangmoon Lee, Cho-Rong Lee, Youngha Lee, Jae-Young Um, Jae Berm Park, Sung Wook Seo, Yoon-La Choi, Sungjoo Kim, Seok-Geun Lee, Murim Choi

**Affiliations:** 1 Department of Biomedical Sciences, Seoul National University College of Medicine, Seoul 03080, Korea; yjyoo@stanford.edu (Y.Y.); darami83@snu.ac.kr (S.L.); ekim8411@snu.ac.kr (C.-R.L.); younghalee@snu.ac.kr (Y.L.); 2 Graduate School, Kyung Hee University, Seoul 02447, Korea; volare1109@khu.ac.kr (S.-Y.P.); minjichoi8466@khu.ac.kr (M.C.); jyum@khu.ac.kr (J.-Y.U.); 3 Samsung Advanced Institute for Health Sciences and Technology, Sungkyunkwan University, Seoul 06351, Korea; ebjo@cnbl.co.kr; 4 Department of Surgery, Samsung Medical Center, Sungkyunkwan University School of Medicine, Seoul 06351, Korea; kw1980.lee@samsung.com (K.W.L.); jaeberm.park@samsung.com (J.B.P.);; 5 Sarcoma Research Center, Samsung Biomedical Research Institute, Seoul 06351, Korea; dphong66@gennbio.com; 6 Department of Orthopaedic Surgery, Samsung Medical Center, Sungkyunkwan University School of Medicine, Seoul 06351, Korea; sungwseo@skku.edu; 7 Department of Pathology and Translational Genomics, Sungkyunkwan University School of Medicine, Seoul 06351, Korea; ylachoi@skku.edu; 8 GenNbio, Seoul 08340, Korea

**Keywords:** liposarcoma, dedifferentiated and well-differentiated form, histones, HMGA2

## Abstract

**Simple Summary:**

Although the genetic event that initiates liposarcoma is relatively well studied, understanding how the tumor progresses and displays differential severities will shed new insights into the diagnosis and prognosis determination of patients with liposarcoma. We analyzed the genome and transcriptome of liposarcoma samples and found that the overexpression of the histone gene cluster is an important determinant for dedifferentiated liposarcoma in predicting genome instability and cell proliferation. The overexpression of histone genes was caused by increased HMGA2 with amplification of chromosome 12q13-14. This dedifferentiated liposarcoma-specific genetic signature may serve as a biomarker for prognosis prediction.

**Abstract:**

Liposarcoma (LPS) is an adult soft tissue malignancy that arises from fat tissue, where well-differentiated (WD) and dedifferentiated (DD) forms are the most common. DDLPS represents the progression of WDLPS into a more aggressive high-grade and metastatic form. Although a few DNA copy-number amplifications are known to be specifically found in WD- or DDLPS, systematic genetic differences that signify subtype determination between WDLPS and DDLPS remain unclear. Here, we profiled the genome and transcriptome of 38 LPS tumors to uncover the genetic signatures of subtype differences. Replication-dependent histone (RD-HIST) mRNAs were highly elevated and their regulation was disrupted in a subset of DDLPS, increasing cellular histone molecule levels, as measured using RNA-seq (the averaged fold change of 53 RD-HIST genes between the DD and WD samples was 10.9) and immunohistochemistry. The change was not observed in normal tissues. Integrated whole-exome sequencing, RNA-seq, and methylation analyses revealed that the overexpressed *HMGA2* (the fold change between DD and WD samples was 7.3) was responsible for the increased RD-HIST level, leading to aberrant cell proliferation. Therefore, HMGA2-mediated elevation of RD-HISTs were crucial events in determining the aggressiveness of DDLPS, which may serve as a biomarker for prognosis prediction for liposarcoma patients.

## 1. Introduction

Liposarcomas (LPSs), which are malignant tumors of adipocyte origin, are the most common type of soft tissue sarcoma [1,2]. Among the four main subtypes, well-differentiated LPS (WDLPS) and de-differentiated LPS (DDLPS) constitute the majority [2,3]. WDLPS still possesses adipocyte characteristics, whereas DDLPS loses them and de-differentiates into actively dividing cells [2,4]. WDLPS and DDLPS often coexist in a patient, but many are discovered isolated, suggesting variable initiation and progression mechanisms [2]. DDLPS typically displays more aggressive clinical features with increased recurrences (~40%) and metastasis (20–30% to the lungs) [2]. Given that they are mostly radio- and chemo-insensitive and have variable immunosensitivity, LPS requires new treatment methods.

Genetically, a focal amplification in chromosome (chr) 12 (12q13-14) is found in all subtypes. The amplified interval includes *MDM2*, *CDK4*, *HMGA2*, and other genes that induce cell proliferation when amplified. This amplification generates ring-formed neochromosomes, forcing the overexpression of oncogenes and epigenetic silencing of passenger genes in the region via selection pressure [2]. Although the initiation process of LPS is relatively well understood [5], the genetic signatures that define WD- or DDLPS still do not explain all the differences. In the 12q13-14 amplification, DDLPS seems to carry more amplified copies than WDLPS. Outside of chr12, additional amplification loci in 1p32 (including *JUN*) and 6q23 (including *ASK1*), are frequently found in DDLPS in a mutually exclusive manner [6,7]. Like other sarcomas, LPS harbor less somatic SNV burdens compared to epithelial-originated tumors, although DDLPS tends to display a heavier mutation burden than WDLPS [8]. A recent study performed genome-based analysis of DDLPS samples and identified three clusters that correlate with clinical outcome [9].

Despite a number of genome-wide attempts to elucidate LPS progression and subtype specification, a clear picture delineating the genetic difference between the two main LPS subtypes is lacking. Here we report genome and transcriptome profiles of 22 WDLPSs and 16 DDLPSs and describe an unexpected overexpression of replication-dependent histone genes (RD-HIST) in conferring malignancy to DDLPS. We subsequently identify a signal cascade that progresses DDLPS toward further malignancy through increased cell proliferation and RD-HIST overexpression.

## 2. Materials and Methods

### 2.1. Subjects and Tumor Samples

Anonymized tumor tissues from 32 patients aged 19–90 years, composed of 15 females and 17 males, who underwent surgery or diagnostic core biopsy were collected with informed consent according to procedures approved by the Ethics Committees at the Samsung Medical Center (SMC) (Figure S1 and Table S1). Primary surgical and core biopsy sarcoma tumor tissues were transported from the operating room and were stored in cold Dulbecco’s Modified Eagle Medium (DMEM) at −80 °C. For immunohistological analysis, a small piece of tissue was excised and fixed in 10% formalin. Age and sex information were not used as covariates unless specified. Patient recruitment and sample collections in SMC were approved by the local research ethics committee IRB (2013-07-122).

### 2.2. Tumor Cell Indexing

The mitotic index was determined in the tumor tissue by counting mitoses in 50 high-power fields (HPF) in the most mitotically active area of the tumor and calculating the average number of mitoses per 10 HPF. This was done manually and cross-validated by another pathologist. The areas of tumors with the most solid growth pattern and highest mitotic activity (if present) were chosen for Ki67 immunohistochemistry. The areas with the highest numbers of Ki67-labeled nuclei (“hotspots”) were labeled for computer-assisted analysis. At least 8 fields were chosen within the previously labeled hotspots to be evaluated at 20× magnification to obtain the percentage of cells that were positive for Ki67. A pathologist reanalyzed the hotspot areas for the presence of inflammatory cells or artifacts.

### 2.3. Whole-Exome Sequencing

Whole-exome sequencing (WES) was performed at Theragen Etex (Suwon, Korea) using 1 µg of genomic DNA extracted from whole blood or a tumor. Each exome was captured using a SeqCap EZ Exome v2 Kit (Roche Sequencing, Madison, WI, USA) or SureSelect Human All Exon V5 (Agilent Technologies, Santa Clara, CA, USA) and sequenced using HiSeq 2500 or HiSeq 4000 (Illumina Inc., San Diego, CA, USA). Paired-end sequencing was performed with read lengths of 74 base pairs (bps). The raw reads were aligned using BWA-MEM software. Variants were called using Samtools and annotated using in-house pipeline and SnpEff. Somatic variants were analyzed on the basis of the significance of differences in the read coverages between matched tumor and blood samples using the two-tailed Fisher’s exact test to produce a list of putative candidates in tumors ranked by *p*-values. Calls were further evaluated by manual inspection of the aligned reads.

### 2.4. Bulk and Single-Cell RNA-Sequencing

An RNA sequencing library was constructed using a TruSeq stranded mRNA kit (Illumina Inc.) with rRNA depletion from 500 ng of total RNA. A total of 150 bp paired-end reads were generated using HiSeq 2500 (Illumina Inc., San Diego, CA, USA), producing a mean of 70M reads per sample. The reads were aligned to the human transcriptome (Gencode V10; GRCh37.p13) using Tophat [10] and expression counts were generated using HTSeq-count [11]. Normalization and filtering were done using the DESeq2 package [12]. Single-cell sequencing of liposarcoma cell lines was performed using an in-house Drop-seq apparatus [13]. 15GS-041 and 18DD cell lines were used for the Drop-seq. They are in-house primary cell lines derived from WDLPS and DDLPS samples, respectively. The 18DD sample was also used in this study. Both WD and DD cell lines were trypsinized for 5 min with 0.25% trypsin-EDTA, spun down at 300× g for 5 min, resuspended in 1 mL of PBS-BSA (0.01% BSA), and then centrifuged at 300× g for 3 min. After removing the supernatant, the cells were resuspended in 1 mL of plain PBS and passed through a 40 μm filter. PBS-BSA (0.01% BSA) was used for making the final dilution. Reads were aligned using STAR (version 2.5.2b) [14]. Cell filtering (e.g., mitochondrial gene expression < 5%), gene selection, dimensionality reduction, and clustering were performed using Seurat [15].

### 2.5. LPS Cell Lines and Patient-Derived 18DDLPS

De-differentiated human liposarcoma cell lines LPS246 and LPS863 were provided by Dr. Dina Lev (MD Anderson Cancer Center, Houston, TX, USA). Well-differentiated human liposarcoma cell lines 93T449 and 94T778 were purchased from American Type Culture Collection (Manassas, VA, USA; #CRL-3043 and #CRL-3044, respectively). The 18DD primary cells were established by the Sarcoma Research Center at SMC. The tumor material was excised aseptically from a 74-year-old female patient and then processed as follows: enzymatic dissociation to single cells using sequential incubation in 300 U/mL collagenase (Sigma-Aldrich, St. Louis, MO, USA) at 37 °C and 100 U/mL hyaluronidase (Sigma-Aldrich) for 2.5 h, 0.25% trypsin/EDTA (STEMCELL Technologies, Vancouver, BC, USA) for 4 min, then 5 U/mL dispase (STEMCELL Technologies) plus DNAse (100 μg/mL, Sigma-Aldrich) for 4 min, before passing through a 40 µm filter. LPS246, LPS 863, and 18DD were cultured in DMEM, and 93T449 and 94T778 were cultured in RPMI-1640 supplemented with 10% heat-inactivated fetal bovine serum and 100U/mL of the antibiotics at 37 °C in a humidified incubator with 5% CO_2_.

### 2.6. Western Blots for Fresh, Frozen, and FFPE Tissues

Tumor tissues for Western blots (~10 µg cell lysates) were acquired in a frozen or FFPE status following the tissue storage condition. Frozen tissues were homogenized using a lysis buffer (Cell Signaling, Danvers, MA, USA, #9803) with 1 mM PMSF (TCI, A2098) and protease inhibitor (Roche). The lysed homogenate was centrifuged and supernatants were collected and stored at −80 °C. FFPE tissues were placed in 1.5 mL tubes and deparaffinized in xylene at room temperature for 10 min. After the deparaffinization, tissues were rehydrated with a series of ethanol solutions (100, 90, 80, and 70%). Then, each tissue was resuspended in a cell signaling lysis buffer containing 1 mM PMSF and protease inhibitor. Each tissue sample was incubated on ice for 5 min and then at 80 °C for 2 h. After incubation, the supernatants were collected and stored at −80 °C.

Primary antibodies for HIST H1.5 (Abcam, ab18208, 1:1000), HIST H2A (Abcam, ab104075, 1:200), HIST H3 (Abcam, ab70550, 1:5000), HIST H4 (Abcam, ab10158, 1:1000), HMGA2 (Abcam, ab97276, 1:1000), and β-actin (Sigma-Aldrich, A5541, 1:10,000) were used, followed by horseradish peroxidase-conjugated anti-mouse or anti-rabbit IgG (1:5000) for 1 h and then visualized using the enhanced chemiluminescence detection system.

### 2.7. Immunohistochemistry

Patient samples were deparaffinized and rehydrated, and antigen retrieval was performed in a citrate buffer. The sections were blocked with 5% goat serum at room temperature for 1 h and then incubated with HIST H1.5 (1:800), HIST H2A (1:50), HIST H3 (1:200), and HIST H4 (1:1000) antibodies at 4 °C overnight. After PBS washing, the sections were incubated with each corresponding secondary antibody using a VECTASTAIN^®^ ELITE^®^ ABC kit (Vector Laboratories, Burlingame, CA), and then the staining was visualized using a 3,3`-Diamin-obenzidine kit (Dako, Ely, Cambridgeshire, UK). Counterstaining was performed with hematoxylin. Negative controls were stained with rabbit IgG in parallel with each batch. Images were taken with an Olympus BX51 microscope (Tokyo, Japan).

### 2.8. Lentivirus Production and Transduction

Each vector expressing *HMGA2* (OriGene; RC214681L1), *HMGA2*-shRNA (Sigma; SHCLNG-NM_003483), or *GFP*-shRNA was produced via co-transfection with pMD2.G and psPAX2 into Lenti-X 293T cells (Clonetech). The control vector pLKO.1 GFP shRNA (Addgene, Watertown, MA, USA, #30323) was a gift from Dr. David Sabatini (Massachusetts Institute of Technology, Cambridge, MA), and the envelope vector pMD2.G (Addgene, #12259) and packaging vector psPAX2 (Addgene, #12260) were provided by Dr. Didier Trono (Ecole Polytechnique Federale de Lausanne, Lausanne, Switzerland). Transfections were carried out using Lipo2000 (AptaBio, Gyeonggi, Korea) according to the manufacturer`s instructions. Culture media were harvested at 48, 72, and 96 h post-transfection; centrifuged at 1500 rpm for 5 min; and filtered using a 0.45 µm syringe filter to remove inadvertently collected cells. 94T778 and LPS246 cells were infected with control lentivirus or lentivirus-expressing *HMGA2*, and LPS863 and 18DD cells were infected with lentivirus-expressing *HMGA2*-shRNA or *GFP* shRNA as a control for 24 h. Every infection with lentivirus was carried out in the presence of 8 μg/mL polybrene. After 24 h, cells were selected with 2 μg/mL puromycin. Every infected and selected cell was used for Western blotting, real-time PCR, MTT assays, colony formation assays, and xenograft tumorigenesis experiments as follows.

### 2.9. Western Blots for HMGA2 Knockdown and Overexpression

LPS246 and LPS863 cells were transfected with lentivirus-expressing *HMGA2* or *HMGA2* shRNA and selected with each antibiotic. Then, the cells were collected and lysed with cell signaling lysis buffer with 1 mM PMSF and a protease inhibitor. Primary antibodies for HIST H1.5, HIST H2A, HIST H3, HIST H4, HMGA2, and β-actin were used for immunoblotting, followed by horseradish peroxidase-conjugated anti-mouse IgG or anti-rabbit IgG (1:5000) for 1 h and visualized using the enhanced chemiluminescence detection system.

### 2.10. Colony Forming Assay

LPS246, LPS863, and 18DD cells were plated in 6-well plates (3000 cells/well) and incubated to allow for colony formation. After 15–20 days, the cells were washed with PBS, fixed with methanol for 20 min, and stained with 0.05% crystal violet for 20 min at room temperature. The number of colonies was counted from the digital images. Each assay was performed in triplicate.

### 2.11. Mouse Xenograft Model and Subcutaneous Transplants

NOD.Cg-*Prkdc^scid^Il2rg^tmlWjl^*/SzJ (or NSG; The Jackson Laboratory, Bar Harbor, ME, USA) was maintained under a specific pathogen-free condition in accordance with the ethical guidelines at the Laboratory Animal Research Center of the Samsung Biomedical Research Institute (IACUC #20160617001). LPS863-shCont and LPS863-sh*HMGA2* cells were suspended in a 1:1 *v*/*v* of cold DMEM:Matrigel (BD Biosciences, San Jose, CA, USA) and were subcutaneously engrafted into 6–8-week-old mice. When the size of the tumors approached 1000 mm^3^ in volume, xenograft-bearing mice were euthanized and subjected to further studies. The tumor volume was measured with a caliper every 2–7 days, and volumes (mm^3^) were calculated using (length × width)^2^ × 0.5. Tumor growth curves are presented as average tumor volume ± SD for each group.

### 2.12. Cell Viability Assays

Cell viability was measured using the 3-(4,5-dimethylthiazol-2-yl)2,5-diphenyltetrazolium bromide (MTT) assay. Cells (1–2 × 10^3^/well) were plated in 96-well tissue culture plates and incubated for 24, 48, and 72 h. After incubation, the culture medium was removed and a fresh medium containing 1 mg/mL MTT was added to each well and incubated for 2 h. The optical density (OD) was measured at 590 nm using a microplate reader (Molecular Devices, San Jose, CA, USA). Cell viability was presented in terms of the OD. Each assay was performed in triplicate.

### 2.13. Real-Time Quantitative PCR (RT-qPCR)

Total RNA was isolated using a TRIzol Reagent solution (Ambion, Waltham, MA) according to the manufacturer’s instructions. Total RNA (1 µg) was reverse transcribed using Maxime^TM^ RT PreMix Kit (iNtRON Biotechnology, Kyonggi, Republic of Korea). Amplification of cDNA was monitored using Sensi FAST SYBR NO-ROX kit (Bioline, Taunton, MA) on a StepOnePlus Real-Time PCR system (Applied Biosystems, Waltham, MA). *GAPDH* was used as an internal control. The primers used for amplifying each gene were as follows: *CDK1* (forward: 5′-GGAAGGGGTTCCTAGTACTGC-3′ and reverse: 5′-CCATGTACTGACCAGGAGGGA-3′), *CDK2* (forward: 5′-GACACGCTGCTGGATGTCA-3′ and reverse: 5′-GAGGACCCGATGAGAATGGC-3′), *CDK6* (forward: 5′-TGGATGTTTGCAGGAGAGCTA-3′ and reverse: 5′-GCTCCTTCTTGGCTCAAGGT-3′), *CDC25A* (forward: 5′-GGCAAGCGTGTCATTGTTGT-3′ and reverse: 5′-AGGGTAGTGGAGTTTGGGGT-3′), and *GAPDH* (forward: 5′-CAAGGCTGTGGGCAAGGT-3′ and reverse: 5′-GGAAGGCCATGCCAGTGA-3′). The quantification data of each gene expression is presented as a fold change relative to the control shRNA-transfected cells.

### 2.14. Statistical Analyses

Data are presented as mean ± standard deviation (SD) from at least three independent experiments in duplicate or triplicate and analyzed for statistical significance using the unpaired Student’s *t*-test. *p* < 0.05 was considered as statistically significant.

## 3. Results

### 3.1. Comparison of WD- and DDLPS Expression Profiles

We surveyed the genome and transcriptome of 22 WDLPS and 16 DDLPS pathology-proven samples (Figure S1). WES analysis of these tumors validated the massive somatic focal copy-number amplifications in the chr12 interval encompassing *MDM2* and *CDK4* (mean of 11.8× for WDLPS and 15.5× for DDLPS; Figures S2–S4 and Table S2). A comparison of the focal chr12 copy-number status and expression profile of genes in the interval revealed low correlations (*R*_WD_ = 0.14, *R*_DD_ = 0.10), suggesting epigenetic silencing of the selected genes (Figure S5) [2]. A mean of 14.1 and 23.5 mutations per tumor for the WDLPS and DDLPS samples, respectively, were observed (Figure S6 and Table S3) [16]. Although DDLPS carried more somatic mutations, no recurrent point mutations were observed, ruling out the possibility that novel gain-of-function variants may function as the main drivers of the tumorigenesis process or subtype differences. Previously reported somatic copy-number alterations (SCNAs) that may explain tumor initiation and progression in DDLPS, such as 1p32 and 6q23, were detected in two tumors each, but these SCNAs were not exclusively found in DDLPS (Figure S2).

**Figure 1:**
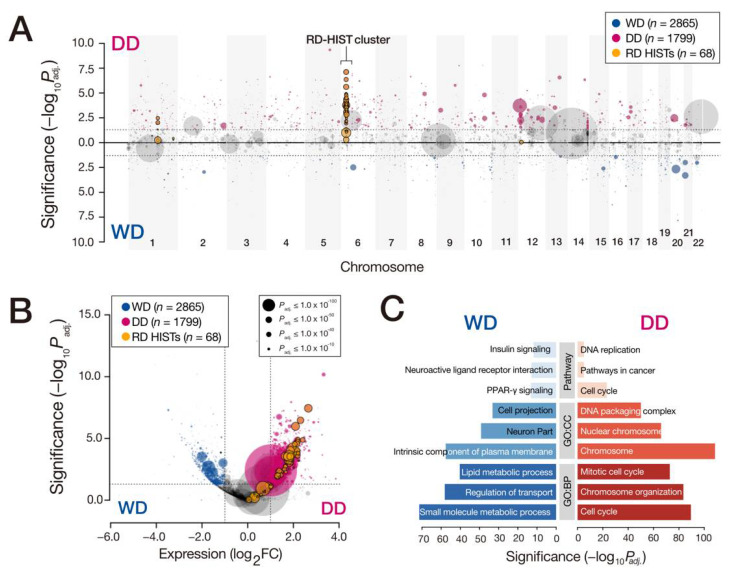
RD-HIST overexpression delineated the difference between WD- and DDLPS. (**A**) Genome-wide view of the differentially expressed genes between 11 WD- and 13 DDLPS tumors, plotted as significances of the differential expression between the two groups. Genes that displayed higher expression in DDLPS are shown in the upper part, whereas higher expressions in WDLPS are in the lower part. Circle sizes denote fold changes in the expression of WD- and DDLPS tumors from the normal adipocyte tissues. (**B**) Volcano plot of the transcripts shown in (**A**), displaying differentially expressed genes in red (up in DDLPS), blue (up in WDLPS), and yellow (RD-HIST genes). (**C**) Gene ontology classes selected from DEGs; *p*-values were from the Wilcoxon rank-sum test, adjusted using the Bonferroni correction method.

We then analyzed the gene expression changes in 11 WDLPS, 13 DDLPS. and 3 normal adipocyte tissues. A total of 4664 transcripts were differentially expressed between WD- and DDLPS, displaying significant enrichments for lipid metabolism (GO:0006629; *P_adj_* = 5.62 × 10^−51^, adjusted using FDR) and PPAR signaling pathway (hsa03320; *P_adj_* = 5.62 × 10^−14^) in WDLPS, as well as cell-cycle (GO:0022402; *P_adj_* = 5.42 × 10^−90^) and chromosome organization (GO:0051276; *P_adj_* = 2.85 × 10^−84^) in DDLPS (Figure 1). We also retained single-cell transcriptome profiles from two patient-derived cell lines (the 15GS-041 line representing WDLPS and the 18DD line representing DDLPS). A total of 412 cells were partitioned into two clusters by their global expression patterns (Figure S7 and Table S4), with DD- and WD-specific expression signatures similar to those from the bulk RNA-seq (Figure 1C). The E2F target and cell-cycle genes were more highly enriched in the DD cluster, whereas the adipogenesis and extracellular structure genes were relatively higher in the WD cluster (Figure S7B,E–G). Expressions of the driver genes in the amplified interval in chr12, *MDM2*, and *CDK4* were similar between the two clusters (Figure S7C).

### 3.2. Overexpression of Replication-Dependent Histone mRNAs in DDLPS

**Figure 2:**
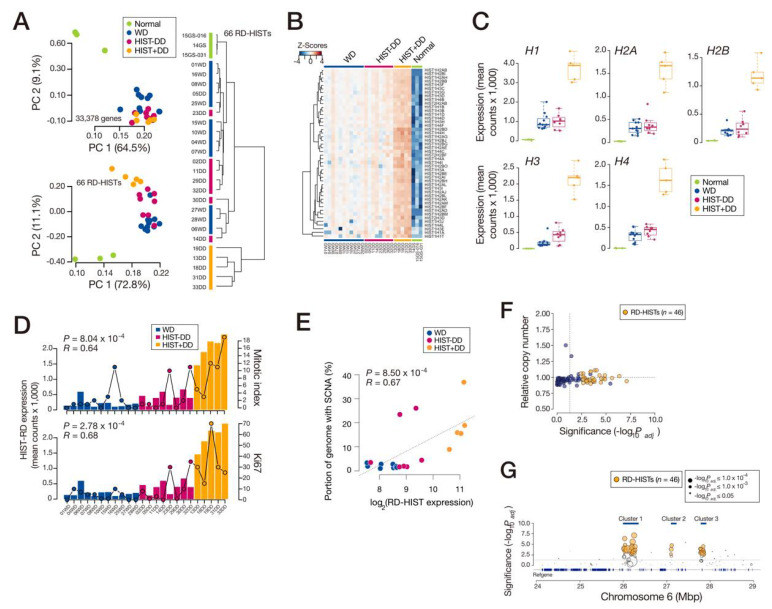
RD-HIST overexpression in a subset of DDLPS samples. (**A**) PCA plot using the entire gene set (upper) and 66 RD-HIST genes (bottom). (**B**) Heatmap of the LPS samples using 66 RD-HIST genes. (**C**) Expression of RD-HIST from RNA-seq transcripts divided by histone types. (**D**) Correlation between RD-HIST expression and cell proliferation indices of the tumor samples. (**E**) Correlation between RD-HIST expression and occurrence of SCNA in the genome. (**F**) Copy-number changes in the genomic interval encompassing the RD-HIST clusters. (**G**) Histone gene-specific differential expression in the cluster. Numbers of samples used for (**A**–**C**): *n* = 27 (WD: 11, HIST-DD: 8, HIST + DD: 5, and normal: 3), (**D**,**E**): *n* = 24 (WD: 11, HIST-DD: 8, and HIST + DD: 5). HIST + DD: histone-positive DDLPS, and HIST-DD: histone-negative DDLPS.

From the comparison of the gene expression profiles, replication-dependent histone (RD-HIST, or canonical histone) mRNAs, clustered in 6p22.2, were highly expressed in DDLPS (48/66 of all RD-HIST genes; Figure 1A,B). A principal component analysis (PCA) and hierarchical clustering demonstrated that the RD-HIST expression pattern represented the differences in tumor status and further differentiated DDLPS into the two subgroups (Figure 2A,B). All RD-HIST families were overexpressed in this subset of DDLPS samples (Figure 2C). Herein, we refer to high-histone DDLPS as HIST+DD (histone-positive DDLPS) and the remaining as HIST-DD (histone-negative DDLPS) tumors. A direct comparison of HIST + DD and HIST-DD tissue expression also pointed to RD-HIST expression as a main differentiating factor (Figure S8). The single-cell analysis also revealed a higher expression of *HIST1H4C* (Figure S7D). TCGA DDLPS set displayed a similar RD-HIST expression pattern in which a small subset behaved like our HIST + DD samples (Figure S9). Furthermore, other cancers in TCGA contained a small subset of tumor samples composed of a high histone expression (cluster #22 in Figure S10).

To validate this observation in the protein level, the expression of selected RD-HIST proteins was examined using Western blot and immunohistochemistry (IHC). The extracts from the HIST + DD samples that were subjected to the RNA-seq analysis and additional frozen and FFPE DDLPS tissues demonstrated an increased expression of histone proteins (Figure S11). Increased RD-HIST had a good correlation with increased tumor cell proliferation (Figure 2D) and an increased propensity for genomic structural variations (Figure 2E), as increased RD-HIST is known to disrupt genome instability and exacerbate tumor progression [17,18]. RD-HISTs achieve S-phase specific gene expression regulation by harboring stem-loop structures in their 3′ end, not in the conventional polyadenylated tail [19,20]. As we performed RNA-seq via poly(A)-capture, the observation of RD-HIST mRNAs from our tumors was unexpected. To validate this observation, we re-extracted RNAs from tumor tissues using a poly(T)-containing kit and performed RT-PCR for histone genes, revealing expression only from the DDLPS samples (Figure S12). Additionally, total RNAs were prepared from a patient-derived DDLPS cell line and subjected to the Oxford Nanopore Direct RNA-seq (Table S5), yielding reads from RD-HIST transcripts with intact polyadenylation (Figure S12). Therefore, the RD-HIST mRNAs were polyadenylated in DDLPS, as previously observed in a carcinogenic condition [21] or terminally differentiated normal tissues [22].

We next tested whether RD-HIST expression still maintained the S-phase-specific increase or whether this phase-specific regulation was ablated. The scRNA-seq analysis and the cell-cycle-synchronized cells expressed RD-HIST in the S-phase (Figure S13). The genomic interval where RD-HIST was located was in a normal copy-number status (Figure 2F), and the increased expression was specific to the RD-HIST genes in the cluster (Figure 2G), suggesting the presence of a histone-gene-specific transcriptional regulatory mechanism.

### 3.3. Overexpression of HMGA2 by the SCNAs

**Figure 3:**
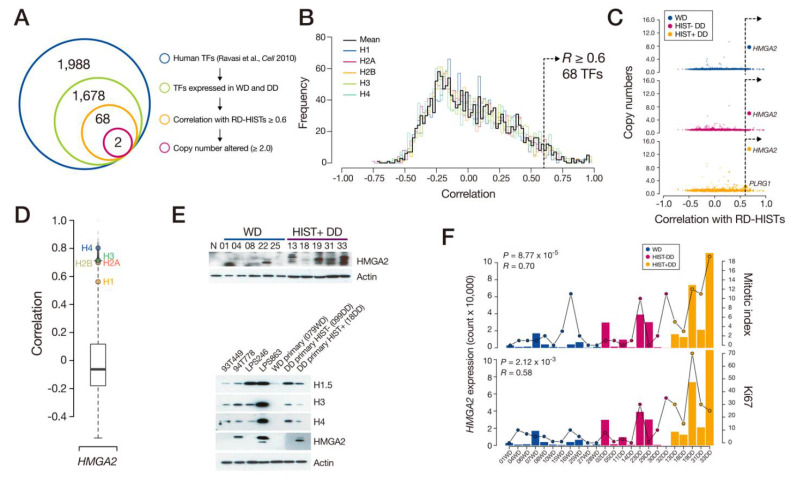
*HMGA2* lay upstream of RD-HIST. (**A**) Identification of TFs showing a strong correlation with RD-HIST and DD-specific somatic amplification, leading to two candidates. (**B**) Correlation distribution of TFs against histone subtypes. (**C**) DD-specific somatic amplification involving the two candidate TFs, namely, *HMGA2* and *PLRG1*. (**D**) Correlation of *HMGA2* against whole genes, displaying histone genes at the top. (**E**) Western blot of HMGA2 and selected histones from tumor samples (up) and various cell lines (bottom). (**F**) Correlation between *HMGA2* expression and cell proliferation indices of the tumor samples.

To address the upstream signal that induced RD-HIST overexpression in HIST + DD, we searched for TFs that were active in the LPS. Among the previously defined 1988 human TFs [23], 84.4% of them were expressed in the WD- and DDLPS samples. In addition, 68 (3.4%) were strongly co-expressed with all members of RD-HISTs in DDLPS samples (Pearson’s correlation *R* > 0.6; Figure 3A,B). As SCNAs are the major driving force underlying DDLPS progression, two TFs, namely, *HMGA2* and *PLRG1*, were selected as they were in the DD-specific SCNAs (>two-copy amplifications). Between the two, *HMGA2* harbored more copy-number amplifications and the gene was located in the chr12 amplification interval (Figure 3C). All five RD-HIST gene families were strongly co-expressed with *HMGA2* in the HIST + DD samples and LPS cell lines (Figure 3D,E). The *HMGA2* copy-number amplification was the most prominent in HIST + DD samples, compared to WDLPS or HIST-DD samples (1.8× and 2.3×, respectively), and *HMGA2* expression was significantly correlated with its copy number (*R* = 0.76, *p* = 1.5 × 10^−5^; Figure S14). *HMGA2* overexpression due to chromosomal rearrangements was strongly linked to tumor malignancy, including LPS [24,25]. Tumors with a high *HMGA2* level also harbored increased proliferation, as represented by the mitotic index and Ki67 (Figure 3F).

### 3.4. HMGA2 Regulated RD-HISTs’ Expression

**Figure 4:**
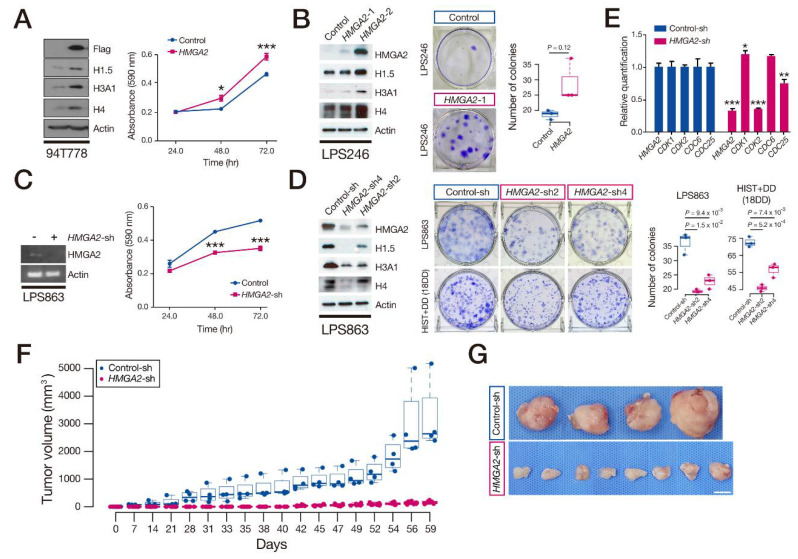
*HMGA2* controls RD-HIST expression, LPS cell proliferation, and tumor size. (**A**) Exogenous introduction of *HMGA2* in 94T778 cells and difference in cell proliferation measured using MTT assays. (**B**) Western blot of selected histones on LPS246—a DDLPS cell line (left) and colony formation assay (right). (**C**) Knockdown of *HMGA2* in LPS863 cells and difference in cell proliferation using MTT assays. (**D**) Western blot of selected histones on LPS863—a DDLPS cell line (left) and colony formation assay (right)—using knockdown of *HMGA2* on LPS863 and 18DD cells. (**E**) RT-PCR quantification of cell-cycle genes in LPS863 cells expressing control-shRNA or *HMGA2*-shRNA. (**F**) Growth of control and *HMGA2*-knockdown cell xenografts in mice. (**G**) Whole-mount images of the xenograft samples in (**F**). Scale bar: 1 cm. (**A**,**C**,**E**) Data presented as the mean ± SD from three independent experiments (*, *p* < 0.005, **, *p* < 0.05, ***, *p* < 0.001).

To test the causality of *HMGA2* overexpression in HIST + DD, we induced *HMGA2* expression to the LPS246 DDLPS cell line, where endogenous *HMGA2* remained relatively low. Increased HMGA2 caused a partial induction of RD-HIST proteins and increased cell proliferation, as measured by colony formation and MTT assays (Figure 4A,B). Next, we delivered the *HMGA2*-shRNA construct to 18DD primary cells and LPS863 DDLPS cells, where *HMGA2* and RD-HIST expressions were relatively high, and observed reduced RD-HIST protein expression and decreased cell proliferation (Figure 4C,D). The expression of a series of S-phase-dependent genes, including *CDK2* and *CDC25*, also decreased in *HMGA2*-deficient DDLPS cells (Figure 4E), consistent with the mRNA-seq profiles. Finally, we evaluated the effects of *HMGA2* overexpression or knockdown in vivo using a xenograft mouse model. LPS863 cells, representing HIST + DD, were transfected with *HMGA2* knockdown or control vectors and were subcutaneously injected into the *NSG* mice [26]. The treatment of knockdown siRNAs exerted an anti-tumor effect in the xenograft model (Figure 4F,G). Therefore, HMGA2 regulated RD-HIST expression and drove tumor progression.

## 4. Discussion

Detailed molecular mechanisms underlying the de-differentiation of LPS and how it gains enhanced malignancy and poor prognosis have remained obscure. We identified a DDLPS-specific RD-HIST overexpression and elucidated upstream molecular factors. We demonstrated that this feature was sufficient for subdividing DDLPS into two groups: HIST + DD and HIST-DD.

The cellular quantity of canonical histones should be tightly maintained [27] and adequately supplied to ensure normal cell division. Animal models representing defective histones cannot develop properly [28,29]. On the other hand, cellular and physiological consequences of overexpressed histones included cellular toxicity, genome instability, and poor cancer prognosis [17,18,30,31]. TCGA data suggest the presence of samples with high histone genes, but their portion in each tumor was not as robust as we observed from DDLPS. Non-canonical histone genes did not display such dramatic changes in expression (data not shown).

In addition, WES and RNA-seq analyses yielded a variety of individual-specific signatures that should not be dismissed. For example, 19DD, a HIST + DD sample, harbored an 8.1-fold copy-number amplification in the chr4 region, including *TDO2*, which encodes a tryptophan 2,3-dioxygenase that converts tryptophan to kynurenine, a metabolite known to be involved in tumor growth and poor prognosis. We demonstrated that the 19DD indeed carried a higher kynurenine/tryptophan ratio (3.8×) compared to other HIST + DD samples (data not shown). Another notable example included 29DD and 30DD, displaying increased immune response gene expression, raising the possibility that these tumors may be favorable to immunotherapy (Figures S8 and S15). These observations underscore the importance of individual-level analysis in addition to seeking global signals to suggest the best therapeutic options tailored for each patient.

A number of clinical parameters of the patients and tumors were assessed for the predictivity of HIST + DD or HIST-DD subtypes, but none turned out to possess enough power except for cell mitosis and Ki67 (Figure 2D). Cellularity displayed a mild correlation (*p* = 0.017, *R* = 0.48). In addition, precise estimation of tumor clonality is important in understanding tumor progression. For example, our single-cell analysis revealed a group of cells with increased E2F target and cell-cycle genes and *HIST1H4C* expression in WDLPS (Figure S7B,E,F), raising the question of whether this group represents a sub-clone in WDLPS that may eventually contribute to HIST + DD. A robust analysis of LPS tissues at a single-cell level would address this question.

Molecular mechanisms underlying the progression of DDLPS into WDLPS remain largely unclear. Although several genetic players have been suggested, the clinical and genetic heterogeneity of the tumors requires analyses of larger sample sets using emerging techniques. *HMGA2* is a tumor-progressing factor in a number of tumor contexts through modulating the critical processes of cancer [32]. In LPS, as the gene is located in the chr12-amplified region along with *MDM2* and *CDK4*, its contribution to LPS progression was evaluated in several studies [33,34,35]. A genome-wide study observed increased *HMGA2* expression in DDLPS compared to WDLPS [33,35], but not in non-malignant lesions with a high proliferation rate [35]. It should also be noted that a pathology study implicated *HMGA2* overexpression with a better prognosis [34].

## 5. Conclusions

Altogether, we identified a genetic signature that differentiates DDLPS into different subtypes, namely, overexpressed histones as a DDLPS-specific factor, and provide a molecular cause of the signature. This result could serve as the rationale for proper patient-specific treatment options for LPS.

## Data Availability

The data presented in this study are available in a reasonable request.

## Supplementary Materials

The following are available online at https://www.mdpi.com/article/10.3390/cancers13133122/s1, Figure S1: Graphical summary of sample information, Figure S2: Somatic copy-number alteration profiles of WD- and DDLPS tumors based on WES, Figure S3: Somatic copy-number alterations of chr12 with fusion events from RNA-seq analysis, Figure S4: Somatic gene fusions on the chr12 interval called from WES and RNA-seq data, Figure S5: Scatter plots displaying correlations between chr12 copy-number fold changes and gene expression levels for WDLPS samples and DDLPS samples, Figure S6: Somatic mutation burden in various tumors extracted from Kandoth et al. (2013), Figure S7: GO analysis on the scRNA-seq data, Figure S8: DEGs between HIST + DDLPS and HIST-DDLPS samples, Figure S9: RD-HISTs expression profile in TCGA sarcoma DDLPS samples displayed in a heatmap and histone-gene-specific bar plots, Figure S10: Histone overexpression in TCGA tumors, Figure S11: Increased RT-HIST protein expressions in DDLPS, Figure S12: Poly(A) tail analysis of LPS RT-HIST transcripts, Figure S13: Cell-cycle analysis of scRNA-seq data, Figure S14: HMGA2 copy-number amplification and correlation with expression, Figure S15: Expression pattern of innate immune response related genes, Table S1: Sample information, Table S2: Somatic copy-number amplification on MDM2 and CDK4, Table S3: Number of somatic mutations of WD- and DD-LPS tumors, Table S4: Summary of single-cell RNA-seq, Table S5: Summary of 18DD MiniON direct RNA-seq analysis.
