# Supplementary material for: Overexpression of Replication-Dependent Histone Signifies a Subset of Dedifferentiated Liposarcoma with Increased Aggressiveness
Source: Cancers (Basel). 2021 Jun 22;13(13):3122. doi: 10.3390/cancers13133122 (PMC8269115; doi:10.3390/cancers13133122)
Supplement: Supplementary file 1 [file cancers-13-03122-s001.zip › cancers-1213981-supplementary.pdf]

Supplementary information

Overexpression of replication-dependent histone signifies a subset of dedifferentiated liposarcoma with increased aggressiveness

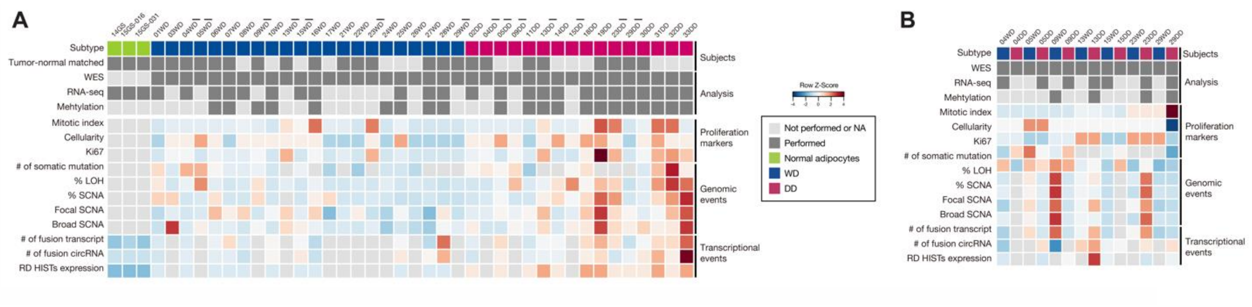

Figure S1. Graphical summary of sample information. (A) Description of all samples and (B) a subset of samples with WD-DD pairs originated from the same patients. Sample IDs with lines denote pairs of WDLPS and DDLPS samples originated from same patients. "Tumor-normal matched" means that both tumor and normal samples were acquired from same donor. WES, whole exome sequencing; RNA-seq, RNA-sequencing; LOH, loss-of-heterozygosity; SCNA, somatic copy-number alteration; circRNA, circular RNA.

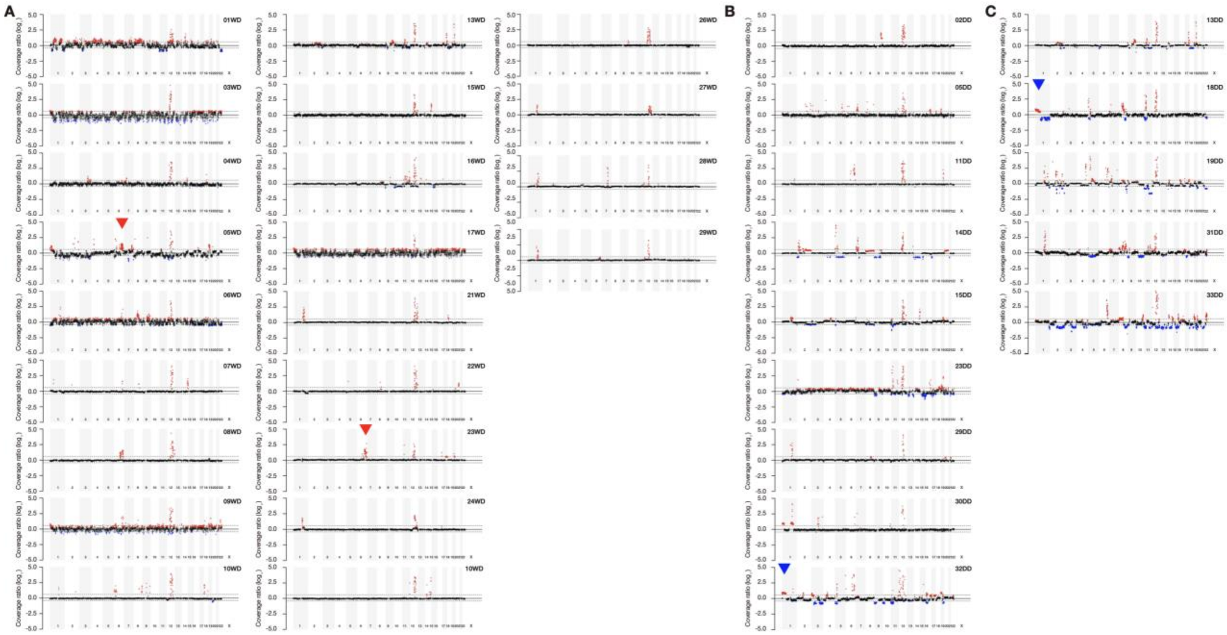

Figure S2. Somatic copy number alteration profiles of WD- and DDLPS tumors based on WES. (A) WD tumors, (B) HIST-DD tumors, and (C) HIST+DD tumors. Blue arrowheads denote 1p32 amplifications (32DD and 18DD) and red arrowheads denote 6q23 amplifications (05WD and 23WD). Copy number alteration in the tumor samples were counted and plotted using copy number status of normal samples as a reference.

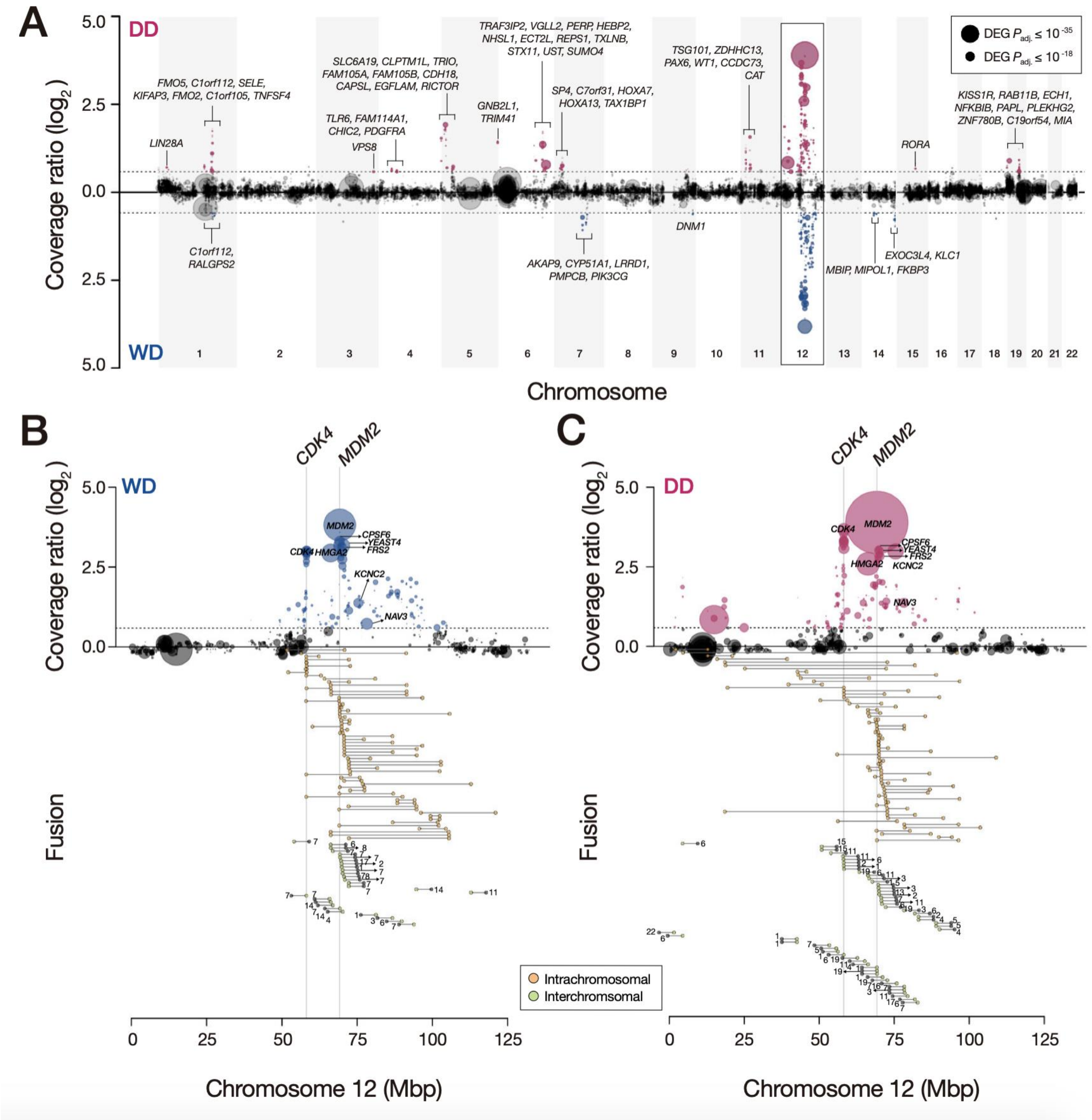

Figure S3. Somatic copy number alterations of chr12 with fusion events from RNA-seq analysis. (A) Genome-wide plot displaying copy number differences of genes in WD- (lower) or DDLPS (upper) tumors. Circle sizes denote *P*-values of differential gene expression. (B-C) Copy number changes, gene expression and RNA-seq based fusion events focusing on the chr12 duplication interval from WDLPS (B) and DDLPS (C) tumors.

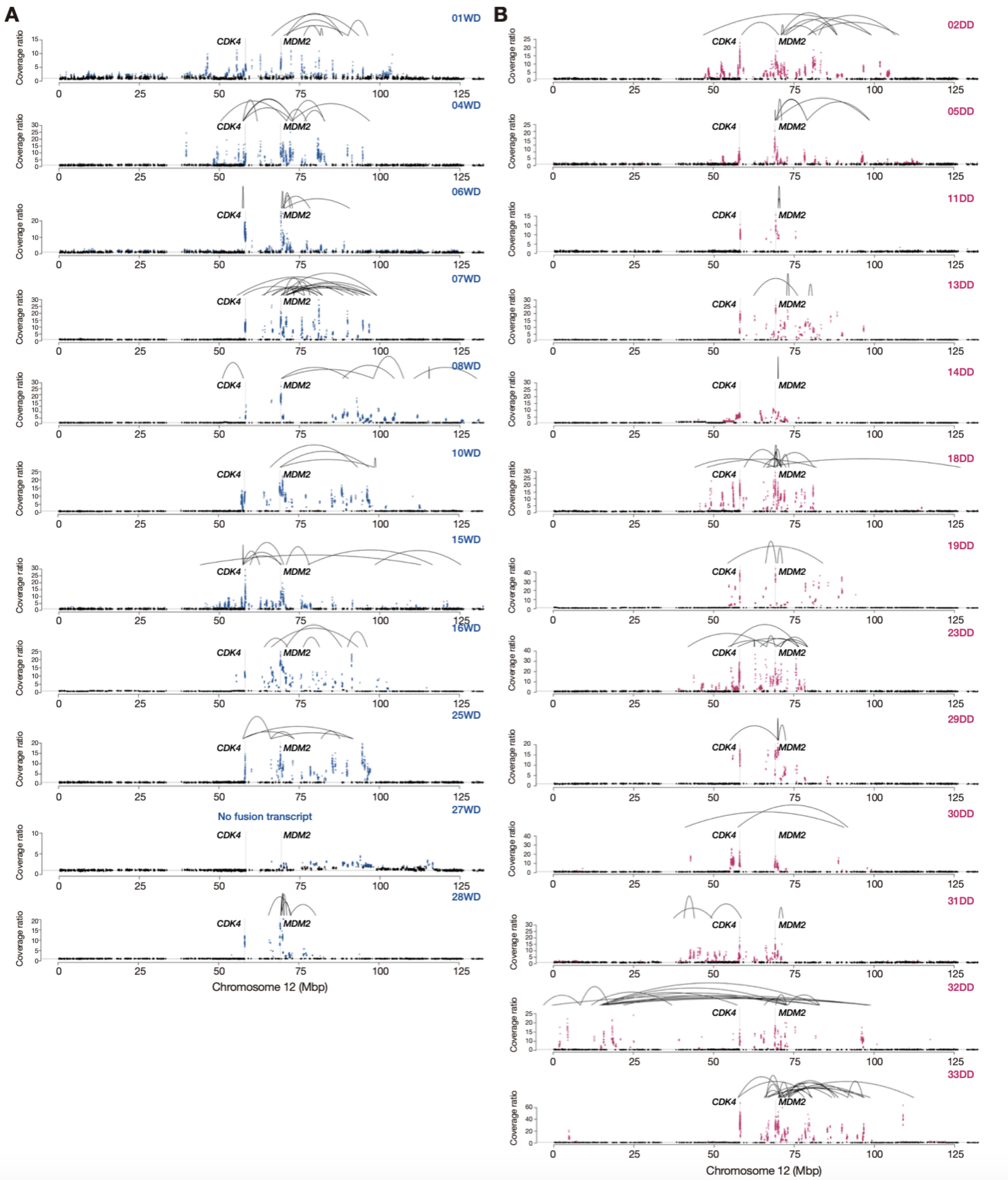

Figure S4. Somatic gene fusions on the chr12 interval called from WES and RNA-seq data. (A) WD tumors, and (B) DD tumors.

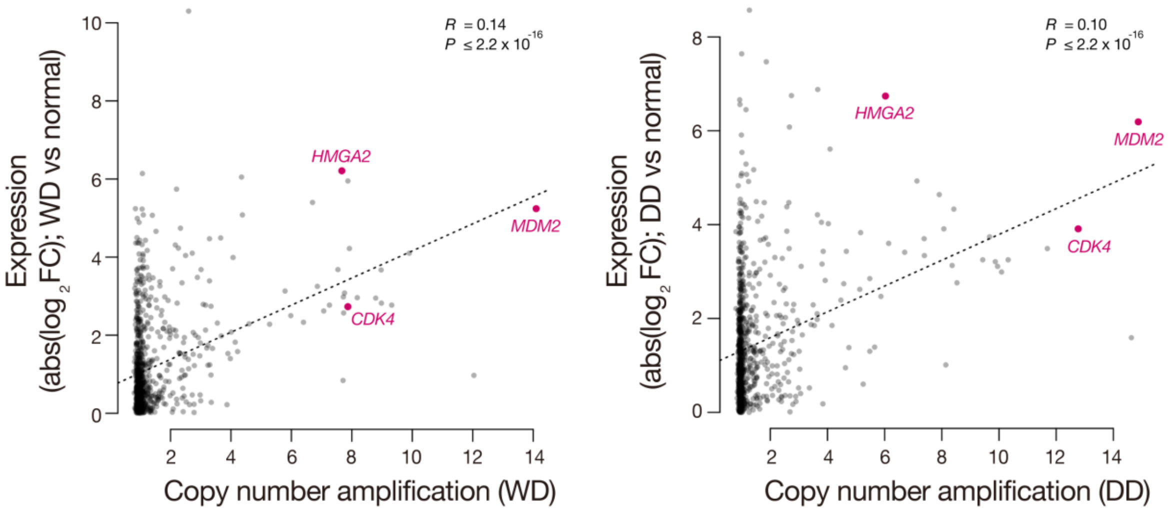

Figure S5. Scatter plots displaying correlations between chr12 copy number fold changes and gene expression levels for WDLPS samples (left) and DDLPS samples (right).

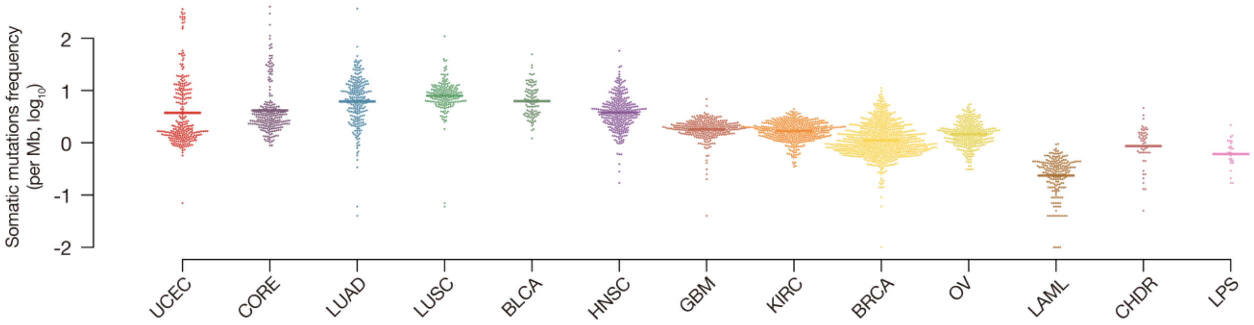

Figure S6. Somatic mutation burden in various tumors extracted from Kandoth *et al.*, (2013) *Nature*.

LPS data used in our study are added on the right for comparison.

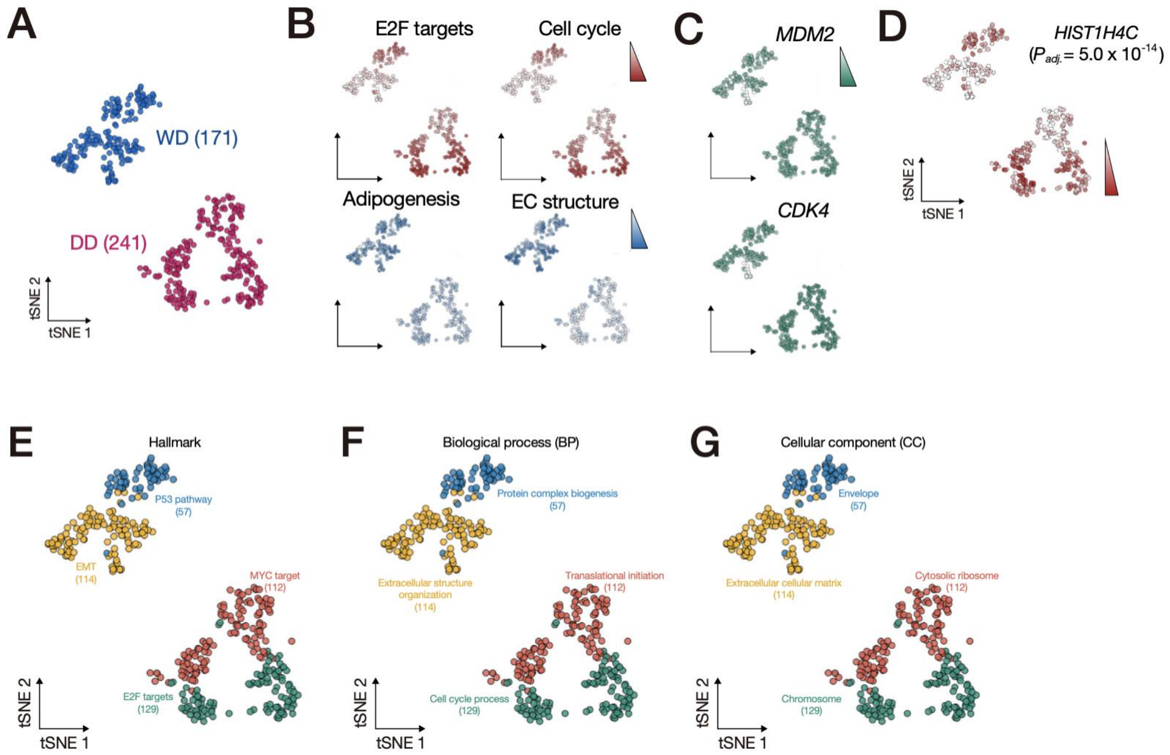

Figure S7. GO analysis on the scRNA-seq data. Analyses of scRNA-seq data using patient-derived WD- and DDLPS cell lines. (A) tSNE plot of WD- and DDLPS cells. (B) tSNE plots with overlaid gene groups that are enriched in each cell line. *P*-values for significant enrichment of each gene group are: E2F targets, $P = 1.4 \times 10^{-114}$; cell cycle, $P = 3.1 \times 10^{-115}$; adipogenesis, $P = 3.4 \times 10^{-60}$; and EC structure, $P = 8.7 \times 10^{-114}$. They were based on a student's t-test. EC structure denotes extracellular matrix structure. (C-D) LPS driver gene and histone gene (*HIST1H4C*) expression. (E-G) Hallmark (E), most prominent biological process (F) and cellular component (G) GOs in each sub-cluster.

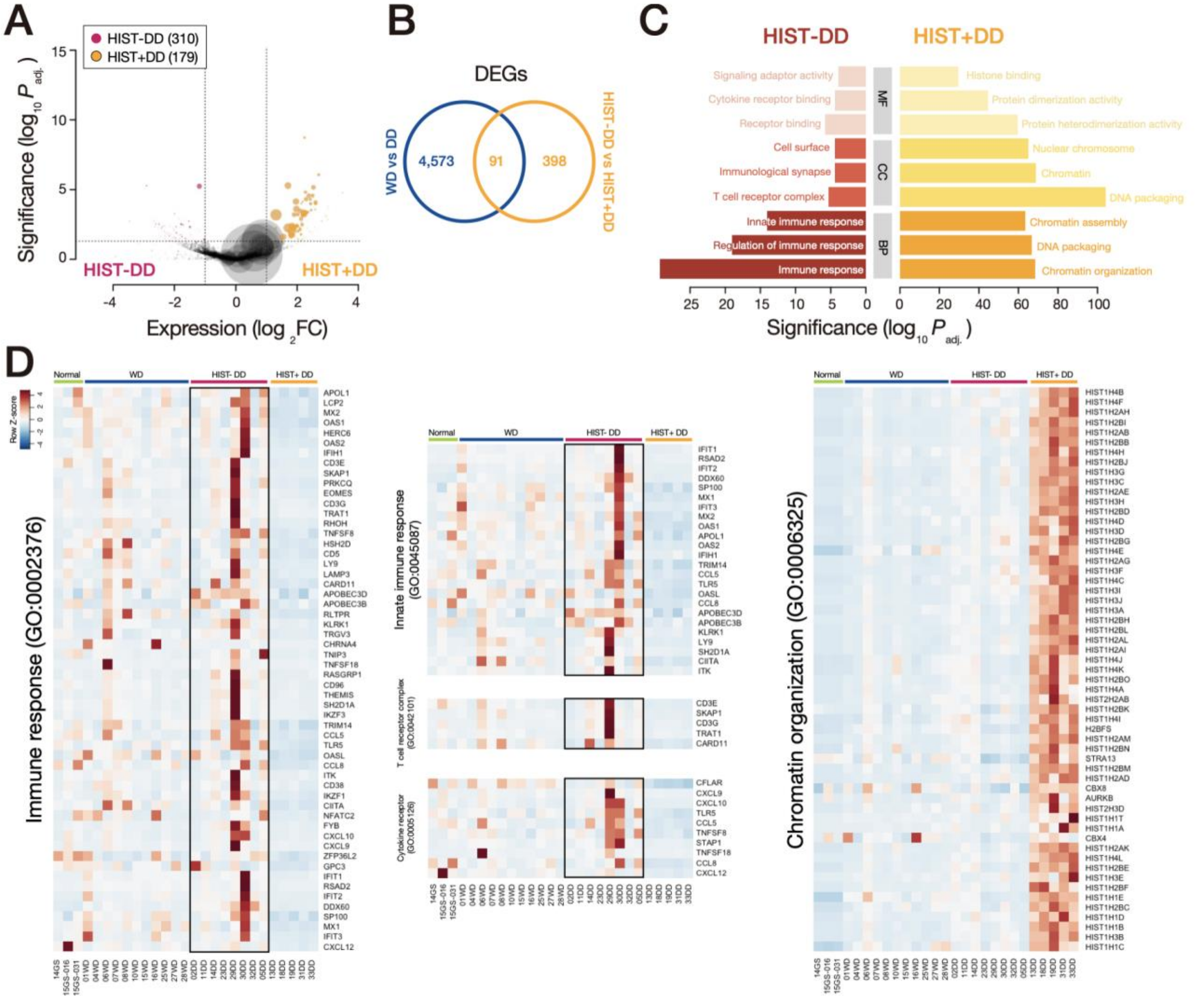

Figure S8. DEGs between HIST+DDLPS and HIST-DDLPS samples. (A) Volcano plots. (B) Venn Diagram of overlapping DEGs. (C) GO enrichment of DEGs from HIST+DDLPS or HIST-DDLPS. (D) Heatmaps of GO term associated genes. HIST+DD group displays increased expression of RD-HIST genes (GO:0006325) whereas HIST-DD group displays increased expression of immune response gene GOs mostly driven by two samples (29DD and 30DD).

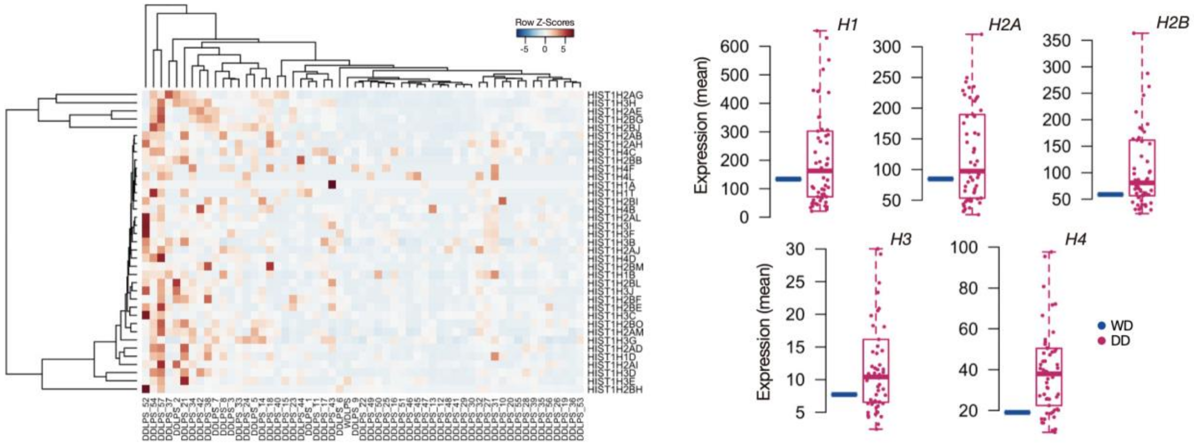

Figure S9. RD-HISTs expression profile in TCGA Sarcoma DDLPS samples displayed in a heatmap (left) and histone gene-specific bar plots (right). There was a single WD sample in the TCGA sarcoma set.

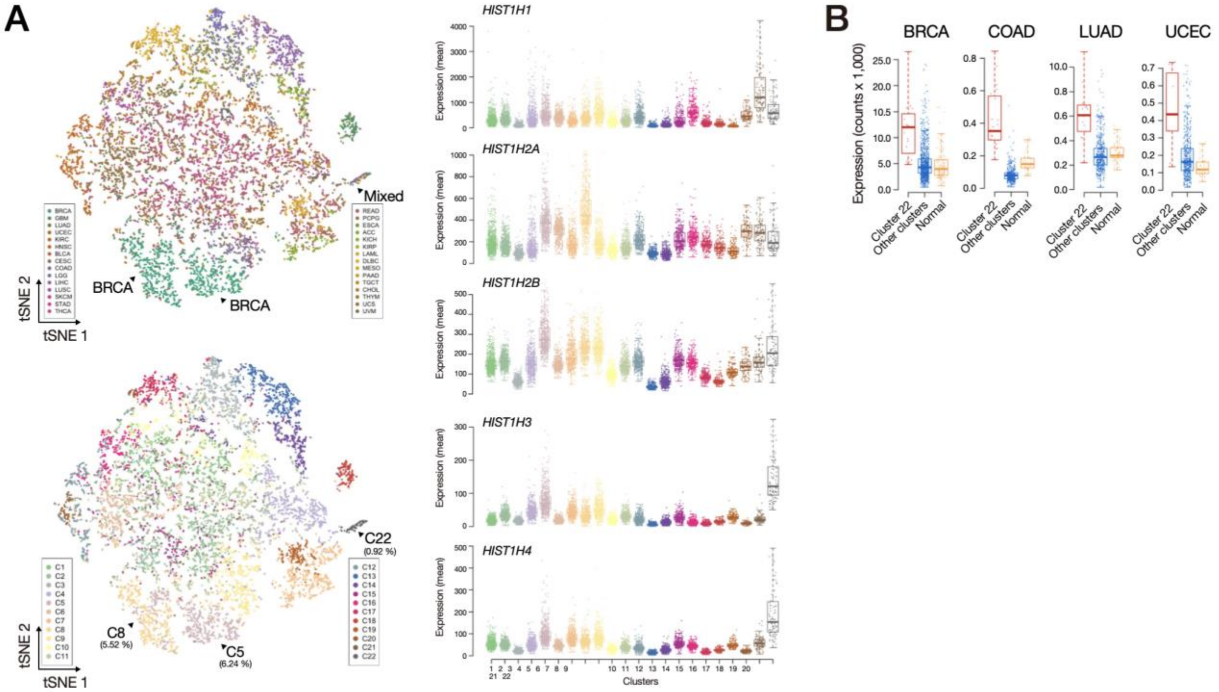

Figure S10. Histone overexpression in TCGA tumors. (A) tSNE plot displaying clustering information of histone genes for major cancer samples. Note C22, a small cluster composed of small numbers (91/9,868 = 0.92%) of various cancer samples shows high histone expression. Box plots of histone expression in different clusters are shown on the right. (B) Comparisons of cluster 22 samples and others in selected major cancer samples (BRCA: breast invasive carcinoma, COAD: colon adenocarcinoma, LUAD: lung adenocarcinoma, UCEC: uterine corpus endometrial carcinoma).

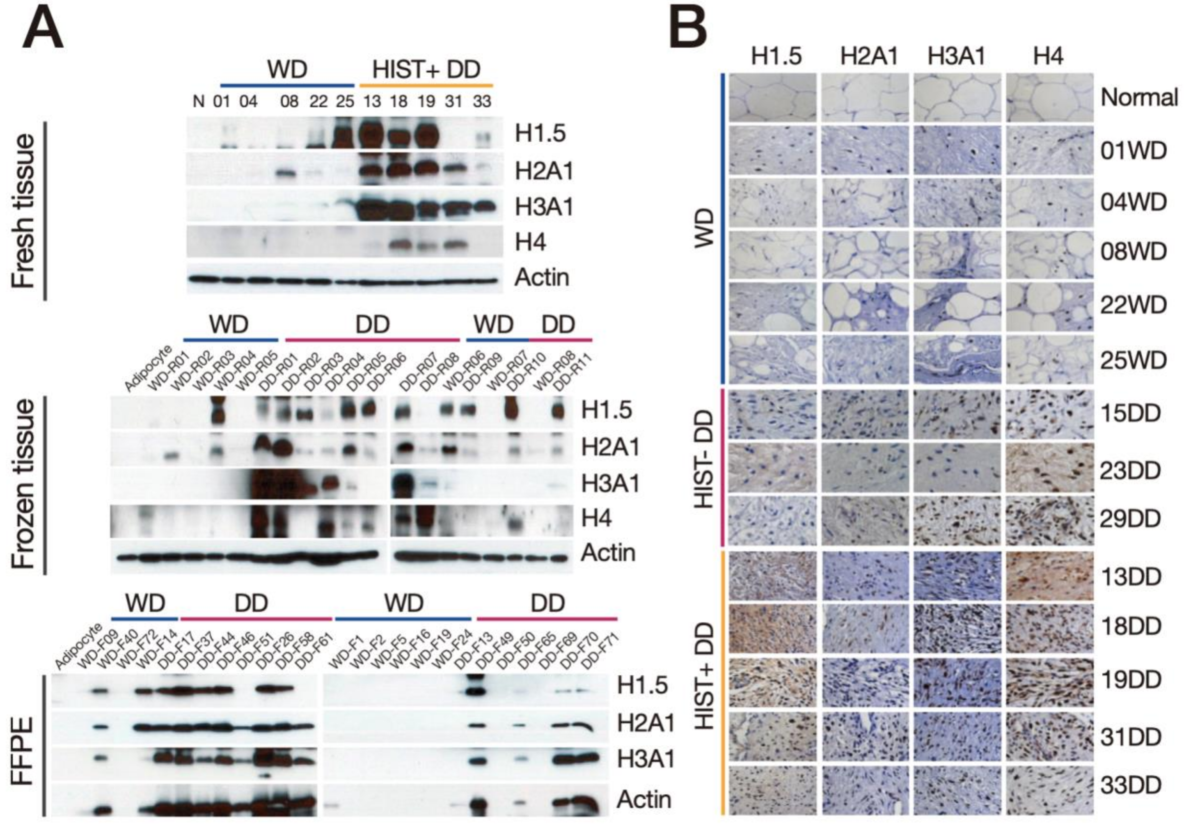

Figure S11. Increased RT-HIST protein expressions in DDLPS. (A) Western blots of histone proteins selected from each subtype using the same samples analyzed by RNA-seq (top), additional frozen (middle) or FFPE (bottom) samples. (B) IHC of histone proteins from the tumors also analyzed by RNA-seq.

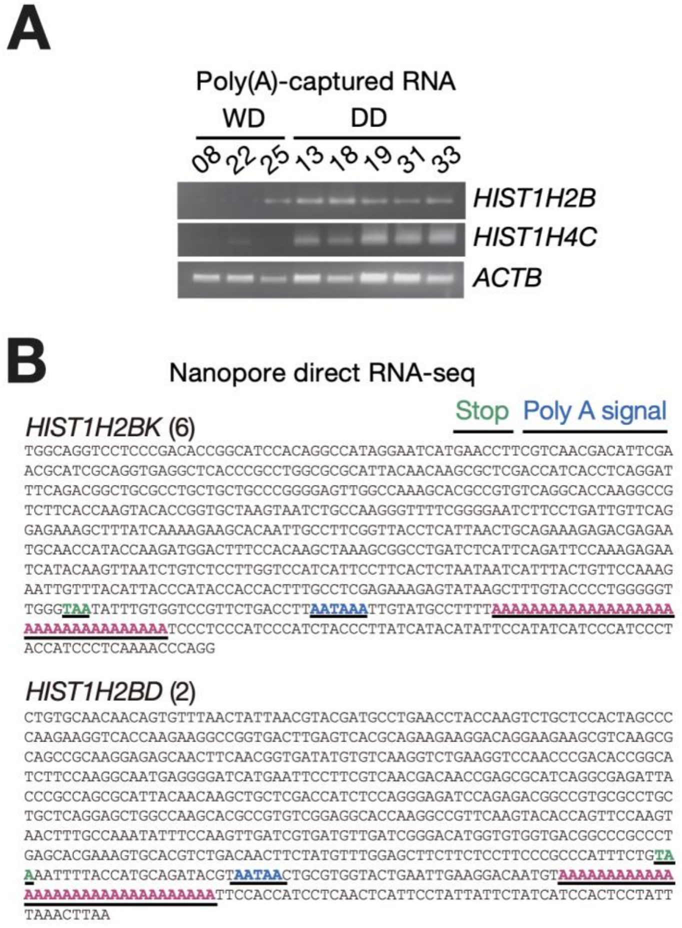

Figure S12. Poly(A) tail analysis of LPS RT-HIST transcripts. (A) RT-PCR results of histone RNAs and loading control (*ACTB*), extracted using poly(T) probes from the tumor tissues. (B) Read sequences aligned to histone genes that were produced from Nanopore direct RNA-seq of DDLPS-derived cells, revealing poly(A) tail sequences.

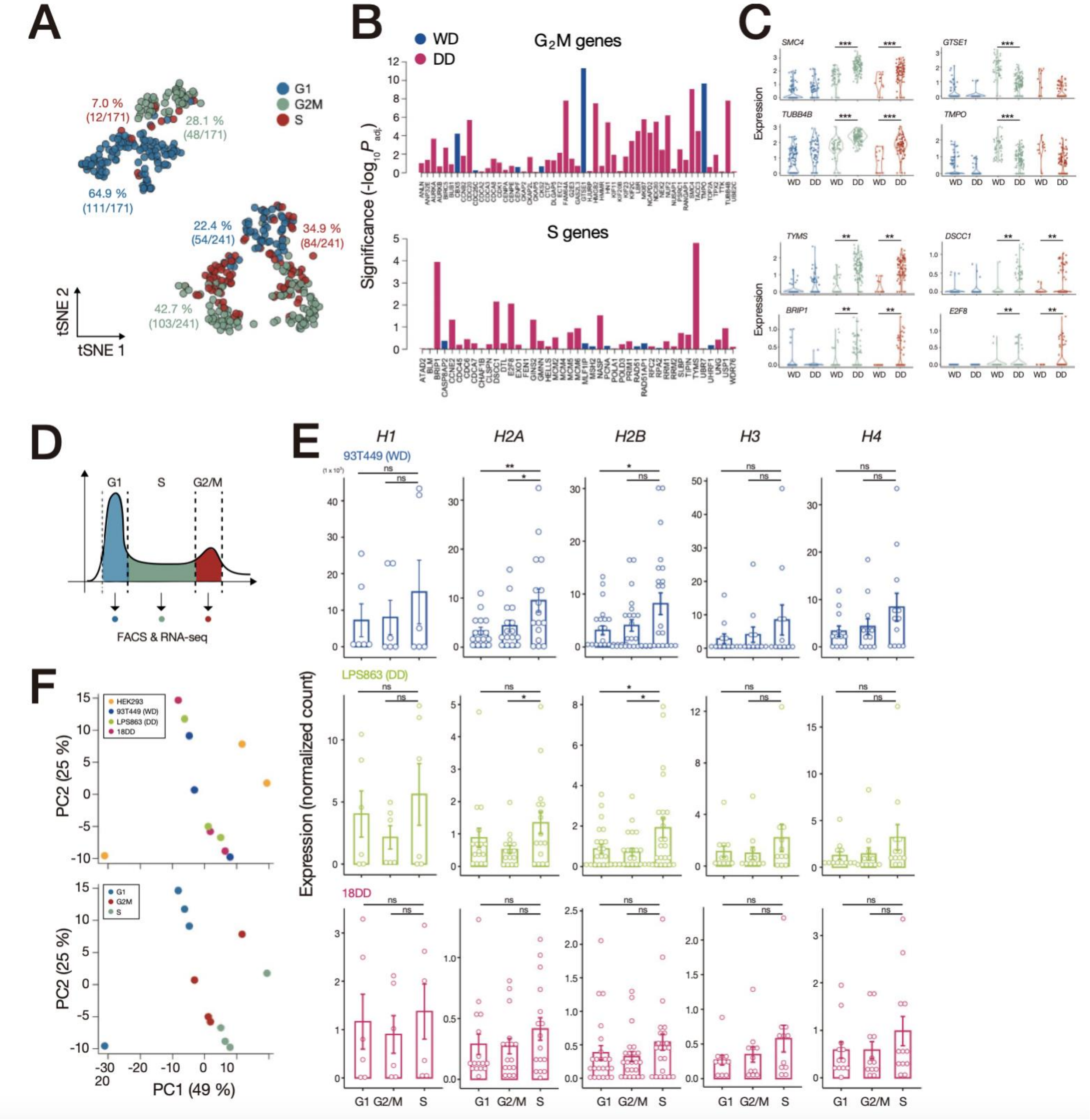

Figure S13. Cell cycle analysis of scRNA-seq data. (A) tSNE plot with cell-cycle assignment. (B) Significance (by student t-test) of increased expression of G2M- (top) or S-phase (bottom) genes in WD- or DDLPS cells. (C) Expression profiles of of G2M- and S-phase genes in WD- and DDLPS cells. (D-F) Cell cycle specific expression of RD-HISTs expression from FACS-sorted RNA-seq data.

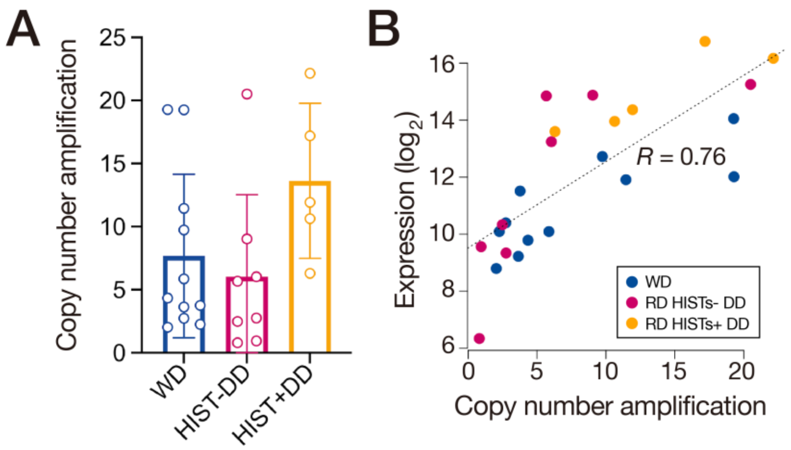

Figure S14. ***HMGA2*** **copy number amplification (A) and correlation with expression (B).**

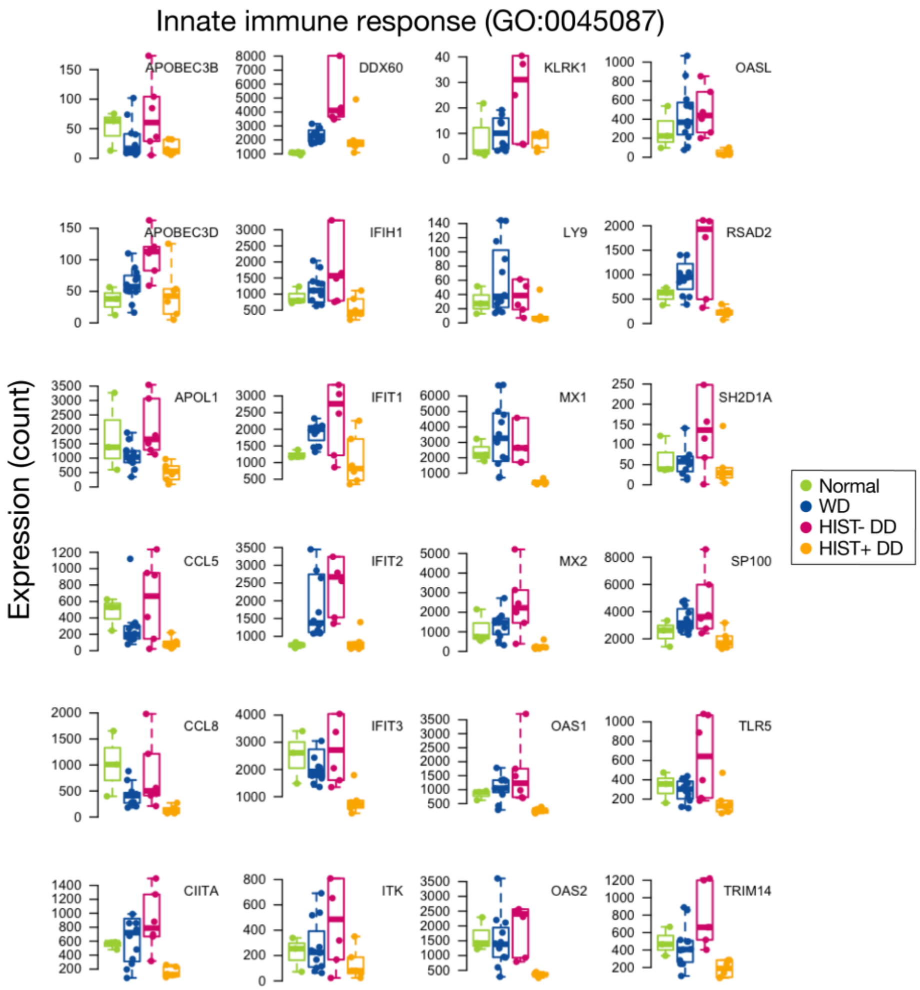

Figure S15. Expression pattern of innate immune response related genes. As shown in Supplementary Figure 8, two WDLPS samples show high expression.

ID	Gender	Age	Primary / Recurrent	Matched normal	Tumor location	Cellularity	Mitosis	MDM2 Histology	MDM2 Copy No.	CDK4 Histology	CDK4 Copy No.	Ki67 Histology	R2 resection	Recurrence (Months)
01WD	M	76	Prim.	O	RPS	1	0	1	N.D	1	N.D	N.D	0	0 (27)
02DD	F	56	Rec.	O	RPS	2	1	1	N.D	1	N.D	8	2	1 (6)
03WD	F	48	Prim.	O	RPS	2	1	1	80	1	80	10	0	0 (27)
04WD	F	49	Prim.	O	RPS	2	1	0	N.D	1	N.D	10	0	0 (26)
04DD	F	49	Prim.	O	RPS	2	1	0	N.D	1	N.D	10	0	0 (26)
05WD	F	55	Prim.	O	RPS	3	1	1	N.D	0	N.D	1	1	0 (26)
05DD	F	55	Prim.	O	RPS	3	1	1	N.D	0	N.D	1	1	0 (26)
06WD	F	68	Rec.	O	RPS	2	1	1	N.D	1	N.D	7	1	1 (60)
07WD	F	57	Prim.	O	RPS	1	1	1	60	1	30	5	0	0 (29)
08WD	M	55	Prim.	O	RPS	2	2	1	25	1	40	5	0	1 (24)
09WD	F	55	Prim.	O	RPS	2	1	1	20	1	40	10	0	0 (20)
09DD	F	55	Prim.	O	RPS	2	1	1	20	1	40	10	0	0 (20)
10WD	M	38	Rec.	O	RPS	2	0	1	40	1	80	1	0	1 (39)
11DD	M	33	Rec.	O	Abdomen	1	0	N.D	N.D	1	N.D	3	1	1 (44)

13WD	M	74	Rec.	X	Mesentery	2	5	N.D	N.D	1	N.D	30	0	1 (10)
13DD	M	74	Rec.	X	Mesentery	2	5	N.D	N.D	1	N.D	30	0	1 (10)
14DD	M	63	Rec.	X	RPS	3	<1	1	16.34	1	N.D	N.D	0	1 (20)
15WD	M	39	Prim.	O	RPS	2	3	1	25	1	N.D	1	0	0 (33)
15DD	M	39	Prim.	O	RPS	2	1	1	25	1	N.D	1	0	0 (33)
16WD	F	82	Rec.	X	RPS	2	11	1	57	1	N.D	10	1	1 (11)
17WD	M	60	Prim.	O	RPS	1	1	1	N.D	1	N.D	N.D	0	1 (22)
18DD	F	75	Prim.	O	RPS	3	3	1	28	1	N.D	15	0	1 (30)
19DD	M	61	Rec.	X	RPS	3	12	1	60	1	60	70	1	1 (14)
21WD	M	37	Rec.	X	RPS	1	1	1	N.D	1	N.D	5	1	1 (39)
22WD	F	44	Prim.	O	RPS	1	0	N.D	23.16	1	N.D	0	0	0 (38)
23WD	M	64	Prim.	X	RPS	2	10	1	>20	1	N.D	30	0	1 (17)
23DD	M	64	Prim.	X	RPS	2	10	1	>20	1	N.D	30	0	1 (17)
24WD	M	66	Prim.	O	Thigh	1	0	N.D	60	N.D	N.D	N.D	0	0 (29)
25WD	F	44	Rec.	O	RPS	3	2	N.D	N.D	1	N.D	5	0	1 (33)

26WD	M	68	Prim.	O	RPS	1	<1	1	25.1	1	N.D	N.D	0	0 (44)
27WD	F	62	Prim.	X	RPS	1	0	0	8.3	1	N.D	N.D	0	0 (34)
28WD	M	49	Prim.	X	RPS	1	0	1	50	1	N.D	N.D	0	0 (37)
29WD	F	39	Rec.	X	RPS	1	0	1	N.D	1	N.D	1	0	1 (103)
29DD	F	39	Rec.	X	RPS	1	0	1	N.D	1	N.D	1	0	1 (103)
30DD	M	58	Rec.	O	RPS	2	2	1	>20	N.D	N.D	10	0	1 (40)
31DD	M	54	Rec.	O	RPS	3	11	1	N.D	1	N.D	30	0	1 (60)
32DD	F	66	Rec.	O	RPS	2	11	1	37.9	1	23.7	35	0	1 (58)
33DD	M	47	Rec.	O	RPS	2	19	1	N.D	1	45.8	25	1	1 (27)

Table S1. Sample information. Cellularity (1: low, 2: intermediates, 3: high); Mitosis (number of mitotic cells per 10 HPFs); MDM/CDK4 histology (0: negative, 1: positive: not determined: N.D); Ki67 histology (percentage (%) of positive cells, not determined: N.D) ; R2 resection (0: grossly complete resection, 1: grossly visible tumor left behind, 2: not detectable).

Table S2. Somatic copy number amplification on *MDM2* and *CDK4*. Fold changes compared to matched blood normal or averaged blood normal sample, for tumors that matched blood sample was unavailable. Mean fold changes of *MDM2* and *CDK4* loci for WD and DD, respectively, indicate 11.8x increase for WD samples and 15.5x increase for DD samples.

Gene	01WD	03WD	04WD	05WD	06WD	07WD	08WD	09WD	10WD	15WD	16WD	22WD	25WD	27WD	28WD	Mean
MDM2	6.82	11.32	9.54	10.15	22.71	15.91	21.18	8.8	13.37	11.48	20.72	15.91	14.06	1.95	17.38	13.42
CDK4	4.87	34.86	7.29	13.52	9.27	8.5	6.05	10.87	6.84	14.7	10.11	6.41	6.47	0.93	11.49	10.15

Gene	02DD	04DD	05DD	09DD	11DD	15DD	18DD	30DD	32DD	31DD	33DD	Mean
MDM2	11.05	11.69	14.77	12.79	14.72	11.72	21.25	11.93	22.66	13.56	31.91	16.19
CDK4	13.51	6.86	6.6	13.39	9.93	14.55	16	18.78	14.8	14.53	34.61	14.86

Table S3. Number of somatic mutations of WD- and DD-LPS tumors.

SAMPLE	Mean	01WD	03WD	04WD	05WD	06WD	07WD	08WD	09WD	10WD	15WD	17WD	22WD	24WD	25WD	26WD
Missense	9.1	16	7	5	24	14	10	11	8	6	11	3	3	9	8	2
Nonsense	0.5	0	1	0	1	0	0	0	1	0	1	1	1	0	1	1
Silent	3.5	9	4	5	8	4	3	2	5	2	3	1	0	2	1	3
Indel	0.6	1	0	0	1	0	1	2	1	1	1	0	0	0	0	1
Exon-intron boundary	0.3	1	0	1	0	1	0	0	1	1	0	0	0	0	0	0
TOTAL	**14.1**	27	12	11	34	19	14	15	16	10	16	5	4	11	10	7

SAMPLE	Mean	02DD	04DD	05DD	09DD	11DD	15DD	18DD	30DD	31DD	32DD	33DD
Missense	15.1	12	15	13	15	4	12	17	9	17	39	13
Nonsense	0.6	2	0	0	0	0	2	0	0	0	0	3
Silent	5.2	3	4	5	11	0	4	6	3	8	12	1
Indel	1.5	0	3	1	0	0	0	3	0	6	1	3
Exon-in boundary	1.0	2	2	0	1	0	2	0	0	1	2	1
TOTAL	**23.5**	19	24	19	27	4	20	26	12	32	54	21

Table S4. Summary of single-cell RNA-seq

	Mean	15GS-041	18DD
Number of input reads (millions)	25.7	23.0	28.3
Average input read length (bp)	85.0	89.0	81.0
Uniquely mapped reads number (millions)	19.4	18.8	19.9
Uniquely mapped reads (%)	76.1	81.8	70.3
Mismatch rate per base (%)	1.3	1.3	1.4
Number of reads mapped to multiple loci (millions)	1.9	1.5	2.3
Reads mapped to multiple loci (%)	7.4	6.6	8.1
Number of reads mapped to too many loci (millions)	0.2	0.09	0.2
Reads mapped to too many loci (%)	0.6	0.4	0.8
Reads unmapped: too many mismatches (%)	0.0	0.0	0.0
Reads unmapped: too short	7.3	4.6	10.1
Reads unmapped: other (%)	8.7	6.7	10.7

Table S5. Summary of 18DD MiniON direct RNA-seq analysis.

18DD MiniON replicates	Replicate #1	Replicate #2	Replicate #3
Number of reads generated	7,573.4	1,752.5	3,695.3
Total base pairs read (millions)	8.8	1.5	4.2
Mean read length (bp)	1,162.7	837.4	1,140.8
Median read length (bp)	1,262.0	828.0	1,112.5
Min read length (bp)	107.5	109.5	22.8
Max read length (bp)	7,402.1	3,939.5	10,715.0
